# Supplementary material for: A live attenuated vaccine to prevent severe neonatal Escherichia coli K1 infections
Source: Nat Commun. 2024 Apr 8;15:3021. doi: 10.1038/s41467-024-46775-x (PMC11001983; doi:10.1038/s41467-024-46775-x)
Supplement: Supplementary file 1 — Supplementary Information [file 41467_2024_46775_MOESM1_ESM.pdf]

Table supplemental 1: Top 10 genes with decreased in vitro expression between *E. coli* K1 E11 WT and *ΔaroA* (in LB)

Name	Log2 fold change	P-value	FDR p-value	Bonferroni
acs	-4,76	0	0	0
cpxP	-4,65	0	0	0
fimA_2	-4,36	0	0	0
fimC	-5	0	0	0
fimD	-5,06	0	0	0
fiml	-4,78	0	0	0
gltA	-3,22	0	0	0
lamB	-6,16	0	0	0
lldP	-4,54	0	0	0
malE	-4,85	0	0	0

Table supplemental 2: Top 10% of the genes with a decreased expression both in LB and DMEM in *E. coli* E11 Δ*aroA* compared to E11WT

aldA	fimD	malF	thiF
aroA	fimF	malG	thiS
btsT	fimG	malK	ydeN
dppB	fimH	metA	ymcF
dppC	fiml	mglC	ypdl
ECS88_RS19780	glpQ	oppB	
fadA	pspO	pgaB	
fadB	inaA	psuG	
fadE	lamB	srlA	
fimA_2	leuA	srlE	
fimC	leuB	sugE	

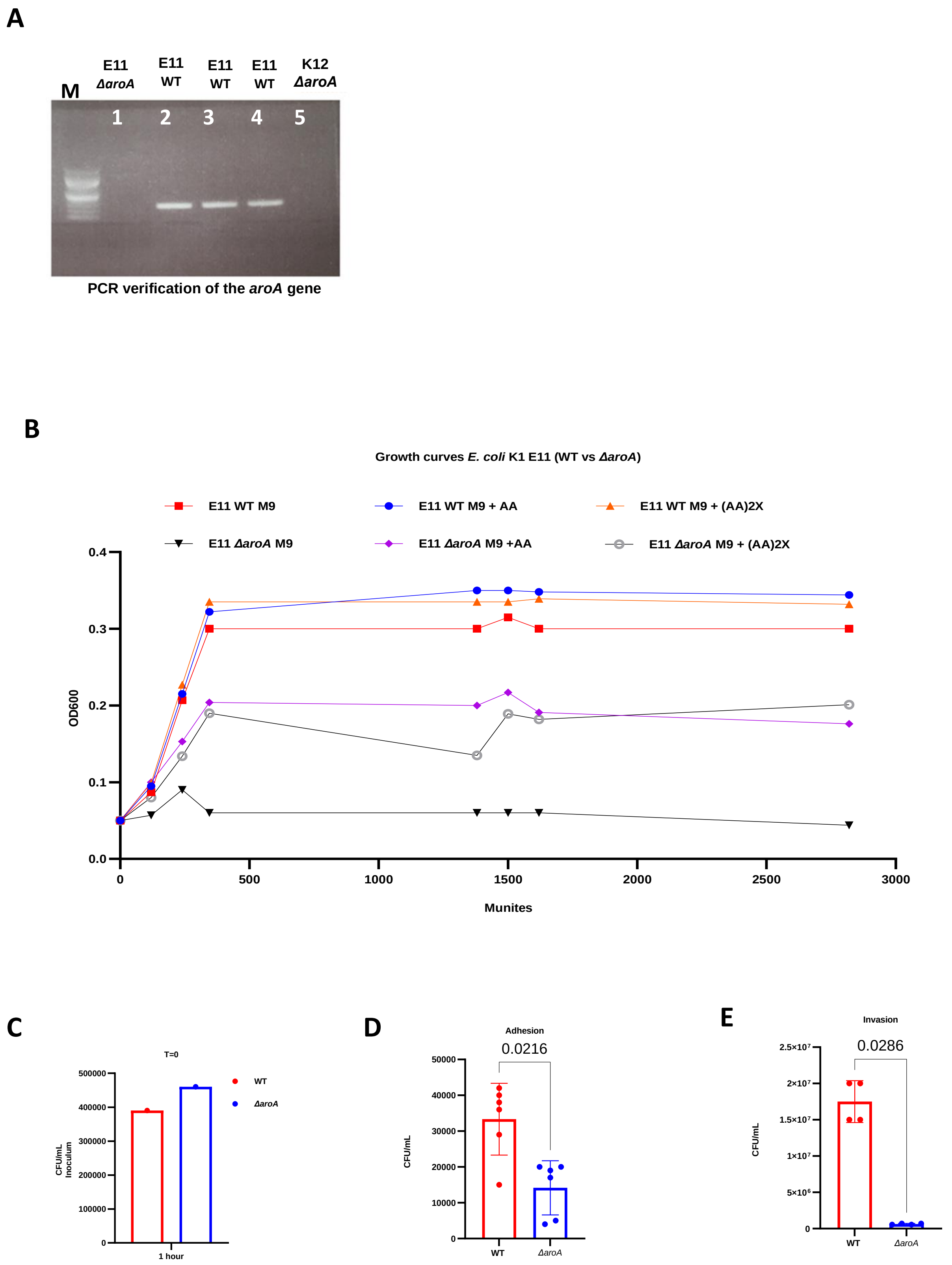

Supplementary figure 1: Confirmatory PCR-based sequencing of the mutant

(A) PCR image of the production of the *E. coli* K1 E11 *aroA* deletion mutant. The *aroA* sequence was partially replaced by the kanamycin resistant cassette at the cleavage sites.

Features of the *aroA E. coli* K1 E11 mutant. Pathway 1: DNA marker. Lane 2: No PCR product for the mutant strain using the *aroA*-Fwd/*aroA*-Rev primer pair. Lanes 3, 4 and 5: the positive control, WT *E. coli* E11. Lane 5: the negative control, *E. coli* K12 *aroA*. (B): Growth curves for *E. coli* E11 WT and *ΔaroA* strains in M9 minimal medium and minimum medium supplemented with amino acids (AA). (C) CFU of inoculum from WT and *ΔaroA* strains, (D) CFU of adhesion to *HeLa* epithelial cells from *E. coli* K1 E11 strains WT vs *ΔaroA* WT vs *ΔaroA* (n=6) and (E) CFU of invasion to Hela epithelial cells from *E. coli* K1 E11 strains WT vs *ΔaroA* (n=6). Error bars represent standard deviations (SD), p-value values indicate significant differences in a two-tailed Mann-Whitney non-parametric test. Source data are provided as a Source Data file.

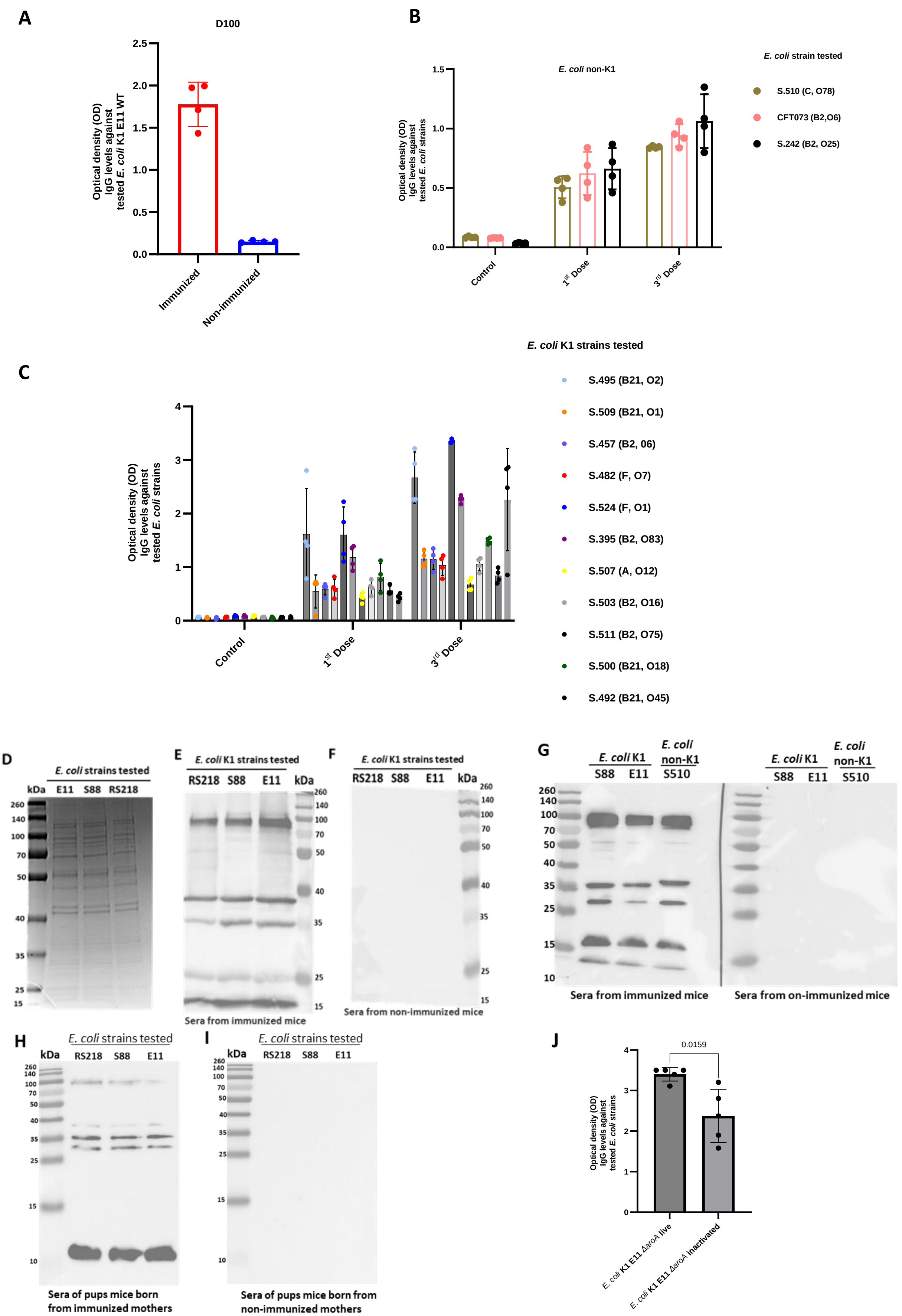
A
D100
Optical density (OD) IgG levels against tested E. coli K1 E11 WT
2.5
2.0
1.5
1.0
0.5
0.0
Immunized
Non-immunized
B
E. coli non-K1
E. coli strain tested
S.510 (C, O78)
CFT073 (B2,O6)
S.242 (B2, O25)
Optical density (OD) IgG levels against tested E. coli strains
Control
1st Dose
3rd Dose
C
E. coli K1 strains tested
S.495 (B21, O2)
S.509 (B21, O1)
S.457 (B2, 06)
S.482 (F, O7)
S.524 (F, O1)
S.395 (B2, O83)
S.507 (A, O12)
S.503 (B2, O16)
S.511 (B2, O75)
S.500 (B21, O18)
S.492 (B21, O45)
Optical density (OD) IgG levels against tested E. coli strains
Control
1st Dose
3rd Dose
D
E. coli strains tested
kDa E11 S88 RS218
E
E. coli K1 strains tested
RS218 S88 E11 kDa
Sera from immunized mice
F
E. coli K1 strains tested
RS218 S88 E11 kDa
Sera from non-immunized mice
G
E. coli K1
E. coli non-K1
S88 E11 S510
Sera from immunized mice
Sera from on-immunized mice
H
E. coli strains tested
kDa RS218 S88 E11
Sera of pups mice born from immunized mothers
I
E. coli strains tested
kDa RS218 S88 E11
Sera of pups mice born from non-immunized mothers
J
0.0159
Optical density (OD) IgG levels against tested E. coli strains
E. coli K1 E11 ΔaroA live
E. coli K1 E11 ΔaroA inactivated

Supplementary figure 2: Antibody responses to other *E. coli* K1 and *E. coli* non-K1 strains responsible for neonatal meningitis in the serum of mice after immunization with *E. coli* K1 E11 *ΔaroA*

(A): Detection of IgG antibodies to *E. coli* K1 E11 100 days after immunization (n=4). (B) IgG response to three non-K1 *E. coli* strains (CFT073 (B2, O6) S242 (B2, O25) and S510 (C, O78) (n=4). (C): IgG responses to other *E. coli* K1 strains with their phylogenetic groups and O serotypes (n=4). The IgG responses were measured using a standard whole-bacterial-cell ELISA. Titers are presented as the OD. Sera were diluted 1/50. (D) SDS-PAGE analyses with Coomassie Blue staining for *E. coli* K1 E11 WT, S88 and RS218 antigens. In the profile, the left lane corresponds to the antigen lane and the left lane corresponds to the molecular weight standard and 6 µg of the protein extracts of the three strains were run in a 12% polyacrylamide gel *E. coli* K1 E11, S88 and RS218. (E and F) Western Blot of *E. coli* K1 antigens, characterization of anti-*E. coli* K1 and non-K1 IgG antibodies in the serum of one immunized mice (E) and non-immunized serum (F). (G) Western blot of *E. coli* antigens, detection of anti-mouse IgG antibodies was performed in the serum of one immunized mouse and control (non-immunized) serum (n=4/group). (H-I) Detection of polyclonal antibodies by Western Blot (H) serums of the pups' mice from immunized mothers and the presence of the control group (I) (serums of the pups mice from non-immunized mothers) (n=4/group). The attenuated strain is much more immunogenic compared to the inactivated strain (p=0.01) (J). Error bars represent standard deviations (SD), p-value values indicate significant differences in a two-tailed Mann-Whitney non-parametric test. Source data are provided as a Source Data file.

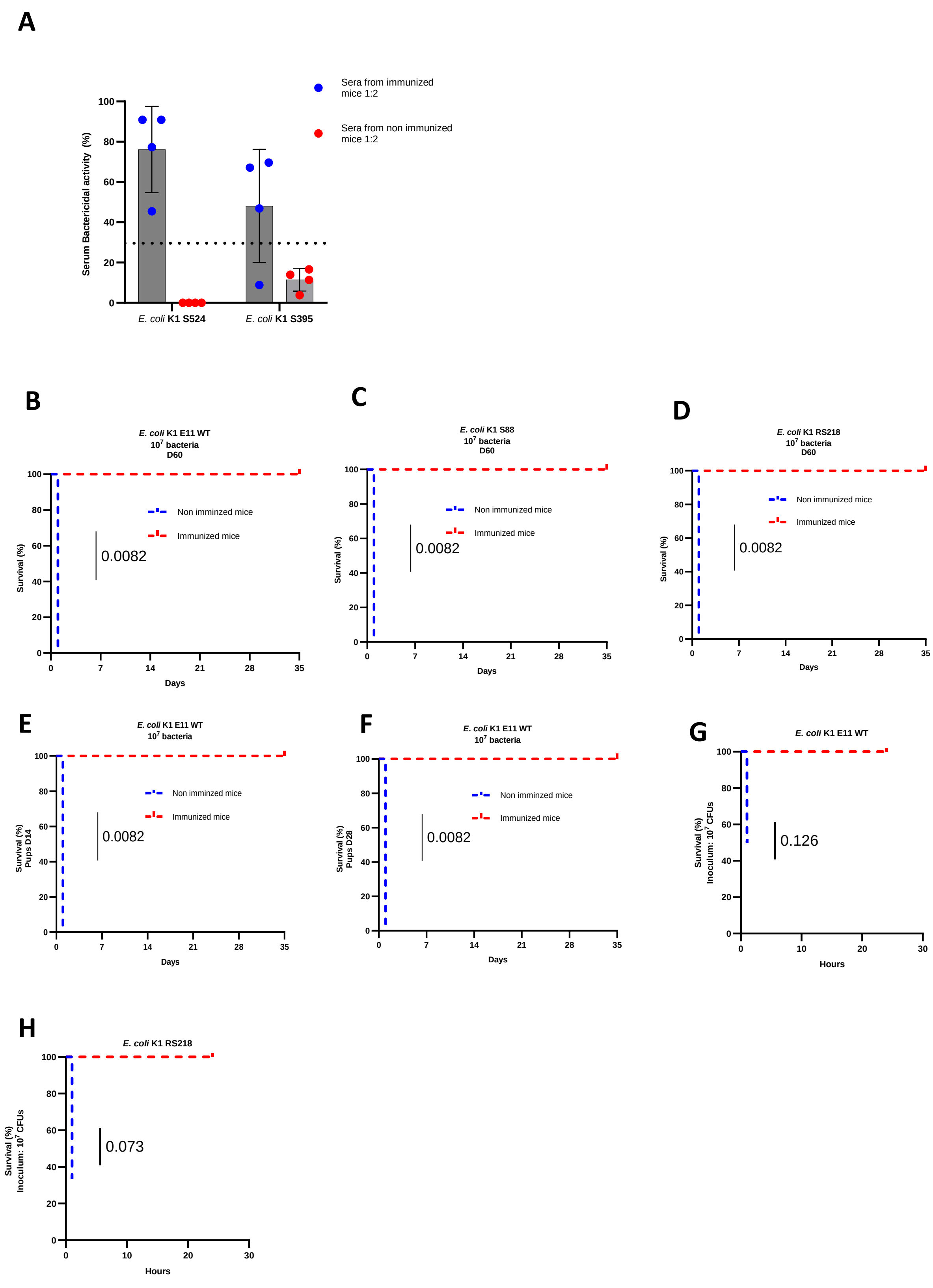

Supplementary figure 3: Protection *in vitro* and *in vivo* conferred by *E. coli* K1 E11 *ΔaroA* attenuated vaccine.

(A): Percentage of bacterial activity obtained with dilution 1:2 of sera from immunized and non-immunized mice (Normal Mice Serum, NMS), (n=4) with the *E. coli* K1 strains S524 and S395, using 3–4-week-old baby rabbit complement.(B-D): 60 days post-vaccination by the IP route, groups of vaccinated female BALB/c mice (n=4 per group) were challenged with lethal doses of 10^7 CFU of *E. coli* K1 E11 WT (B), S88 (C) and RS218 (D) by the IP route. Protection of groups of 14-day-old (E) and 28-day-old (F) pup's mice to immunized mothers exposed to a lethal dose of 10^7 CFU of *E. coli* K1 E11 WT by the IP route (n=4/group). (G-H): Protection of groups of three-day-old pups' mice from immunized mothers exposed to a lethal dose of 10^7 CFU of *E. coli* K1 E11 WT (G) and RS218 (H) by gavage route (n=4/group). Error bars represent standard deviations (SD). P-values indicate significant and non-significant differences in a Mantel–Haenszel log-rank test. Source data are provided as a Source Data file.

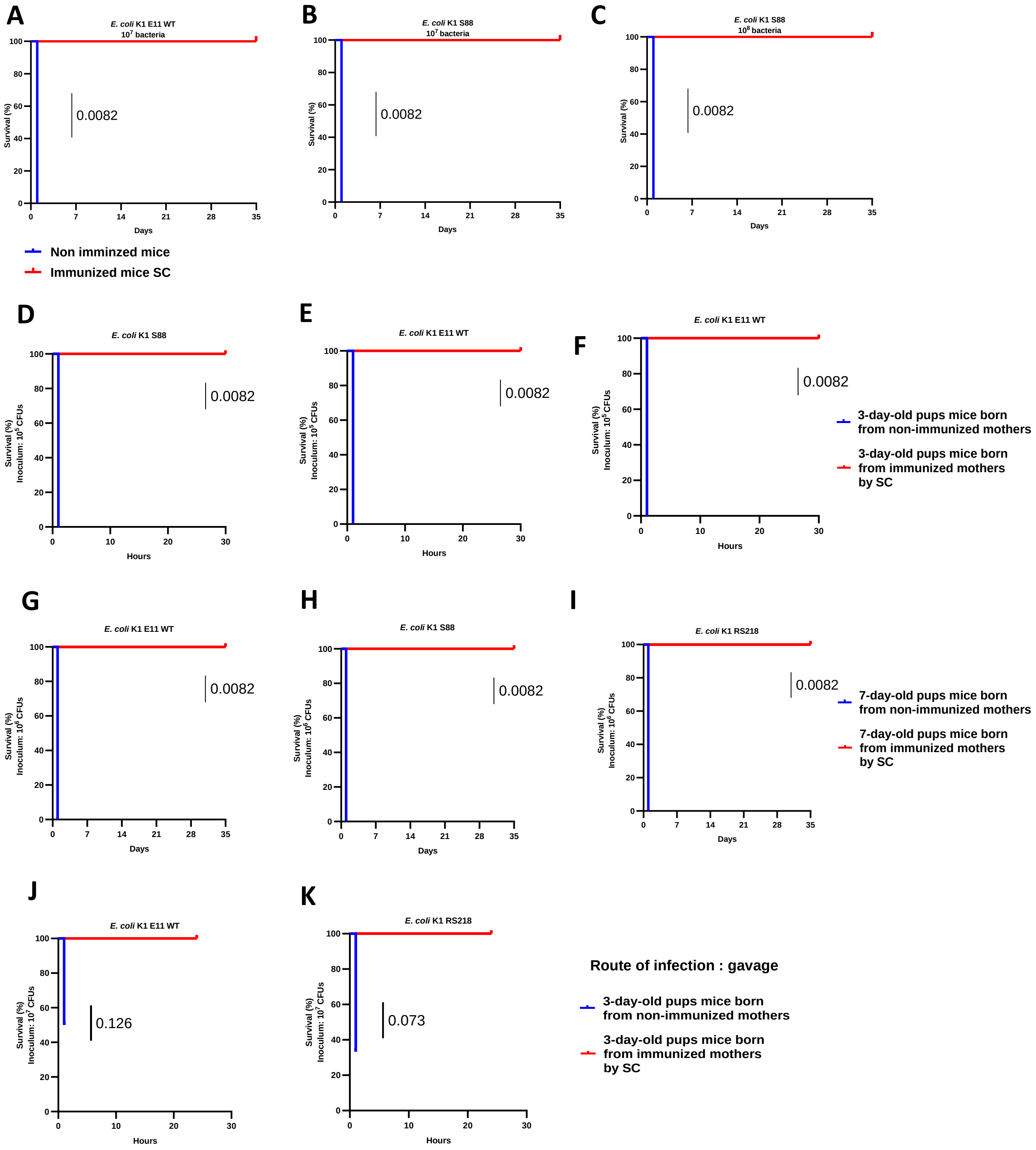

Supplementary figure 4: *In vivo* projection of pups from mothers immunized by the subcutaneous route with the live attenuated *ΔaroA* vaccine.

(A-C): On day 7 post-vaccination SC, groups of vaccinated female BALB/c mice (n=4 per group) were challenged with lethal doses of 10^7 CFU (A) of *E. coli* K1 E11 WT, 10^7 CFU (B) of *E. coli* K1 S88, 10^7 CFU (C) of *E. coli* K1 RS218. (D-F): Three days after delivery, groups

of four pups born to mothers immunized by the SC route and non-immunized female mice were injected intraperitoneally with 10^5 CFU of *E. coli* K1 S88 (D), 10^5 CFU of *E. coli* K1 RS218 (E), and 10^5 CFU *E. coli* K1 E11 WT (F). (G-I): Seven days after delivery, groups of four pups born to mothers immunized by SC and non-immunized females were also given 10^6 CFU *E. coli* K1 E11 WT (G), 10^6 CFU *E. coli K1* S88 (E) and 10^7 *E. coli* K1 RS218 (F). (J-K): Protection of groups of three-day-old pups mice born to immunized mothers exposed to a lethal dose of 10^7 CFU of *E. coli* K1 E11 WT (J) and RS218 (K) by gavage route. Protection was assessed for 24 hours for three-day-old pups' mice and 35 days for 7-day-old pups' mice. P-value indicate significant and non-significant differences in a Mantel–Haenszel log-rank test. Source data are provided as a Source Data file.

A

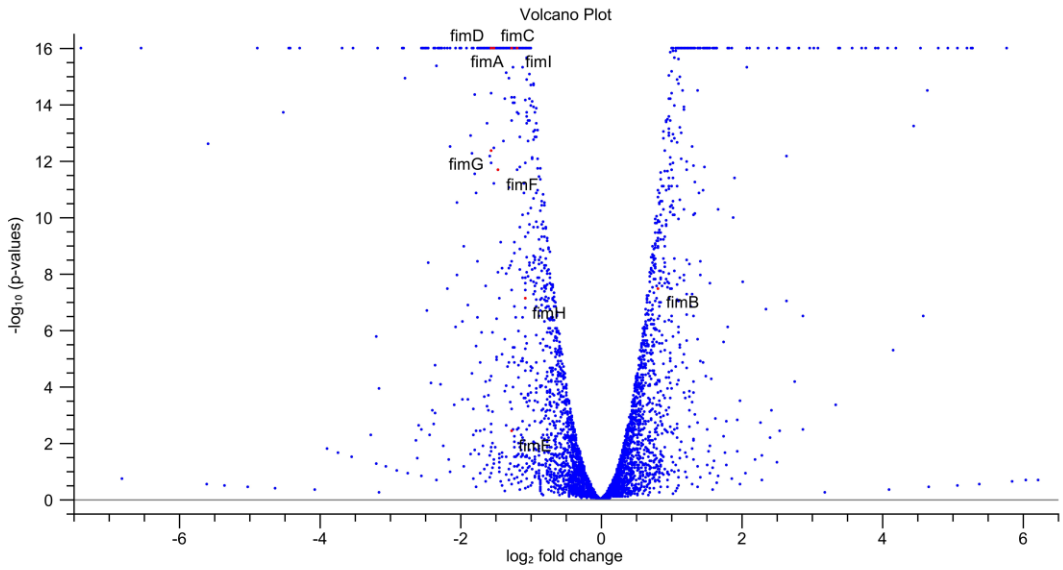

B

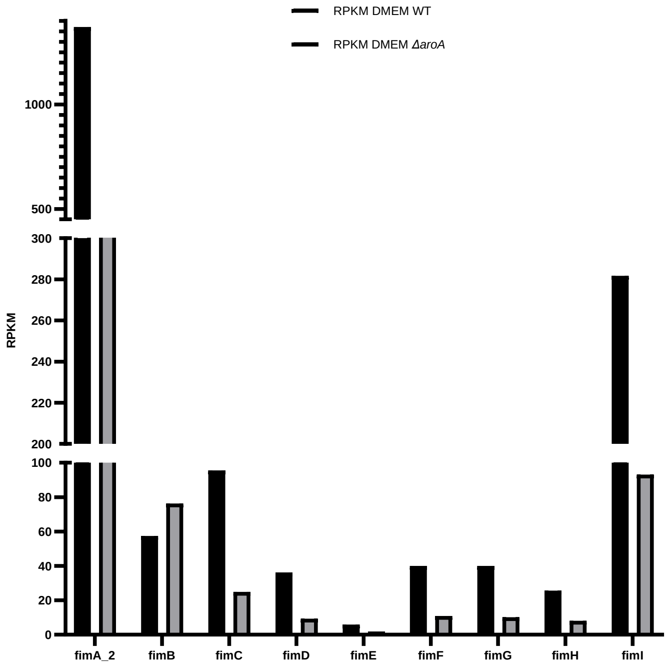

Figure supplemental 5: Comparison of the gene expression between *E. coli* K1 E11 WT and *D*aroA grown in DMEM

(A): RNAseq of *E. coli* E11 WT and *E. coli* E11 *Daro*A grown in DMEM (n=4).

Genes encoding for genes *fim*ABCDEFGHI (red dots) are annotated in the volcano plot: scatterplot showing statistical significance (P values, Y axis) versus magnitude of change (fold change, X axis).

(B): Detailed evolution of the gene expression for the genes *fim*ABCDEFGHI encoding the Type 1 Fimbriae in DMEM between *E. coli* E11 WT and *E. coli* E11 *Daro*A (n=3). Source data are provided as a Source Data file.

Unframed images of all the gels and blots in Supplementary Figure 2

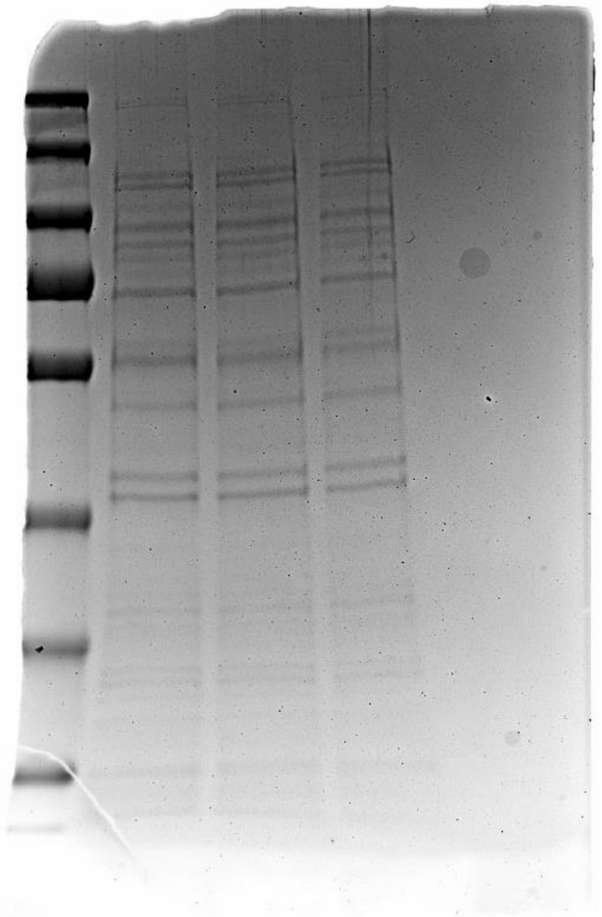

SDS PAGE-Gel

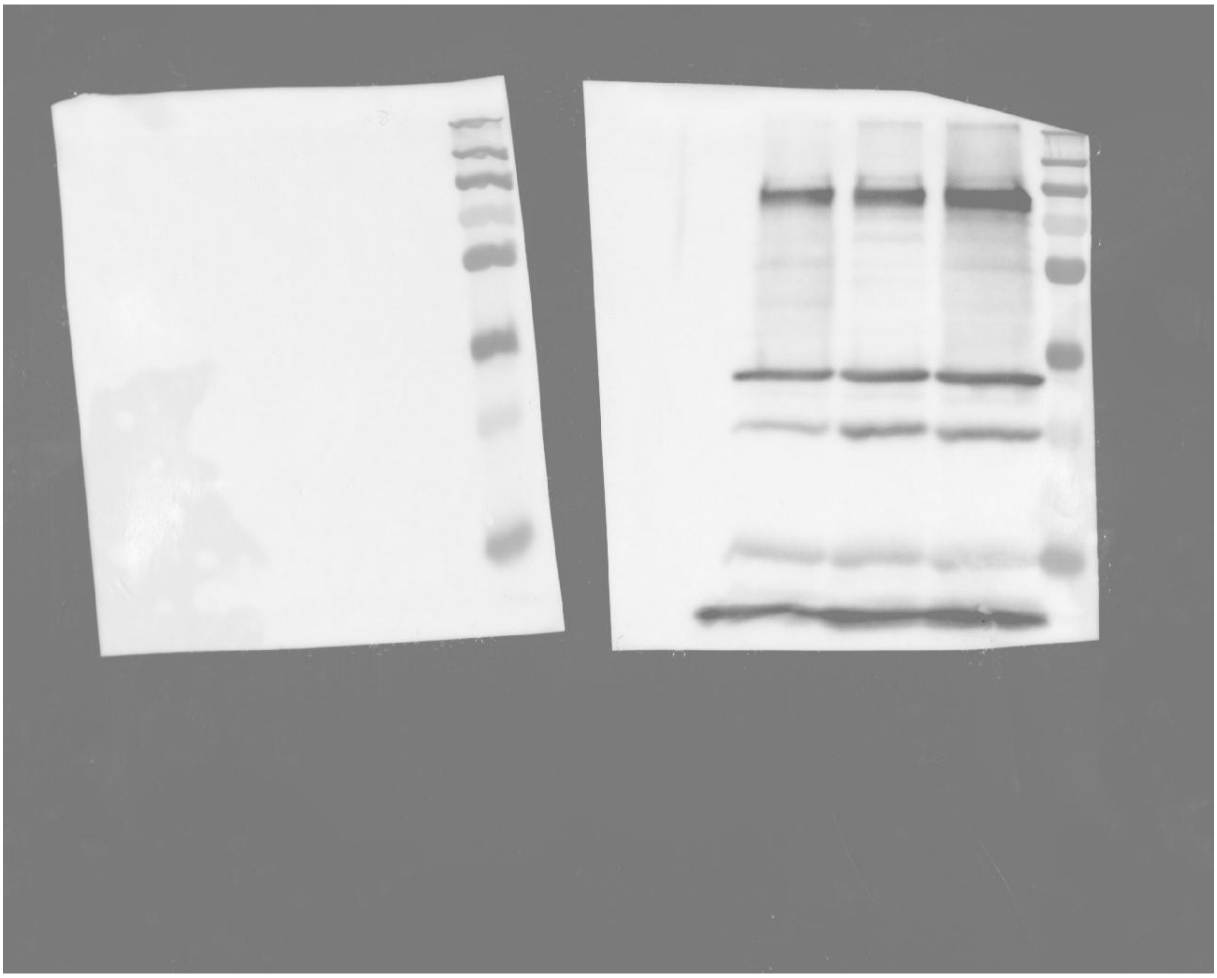

(E and F) Western Blot of *E. coli* K1 antigens, characterization of anti-*E. coli* K1 and non-K1 IgG antibodies in the serum of one immunized mice (E) and non-immunized serum (F)

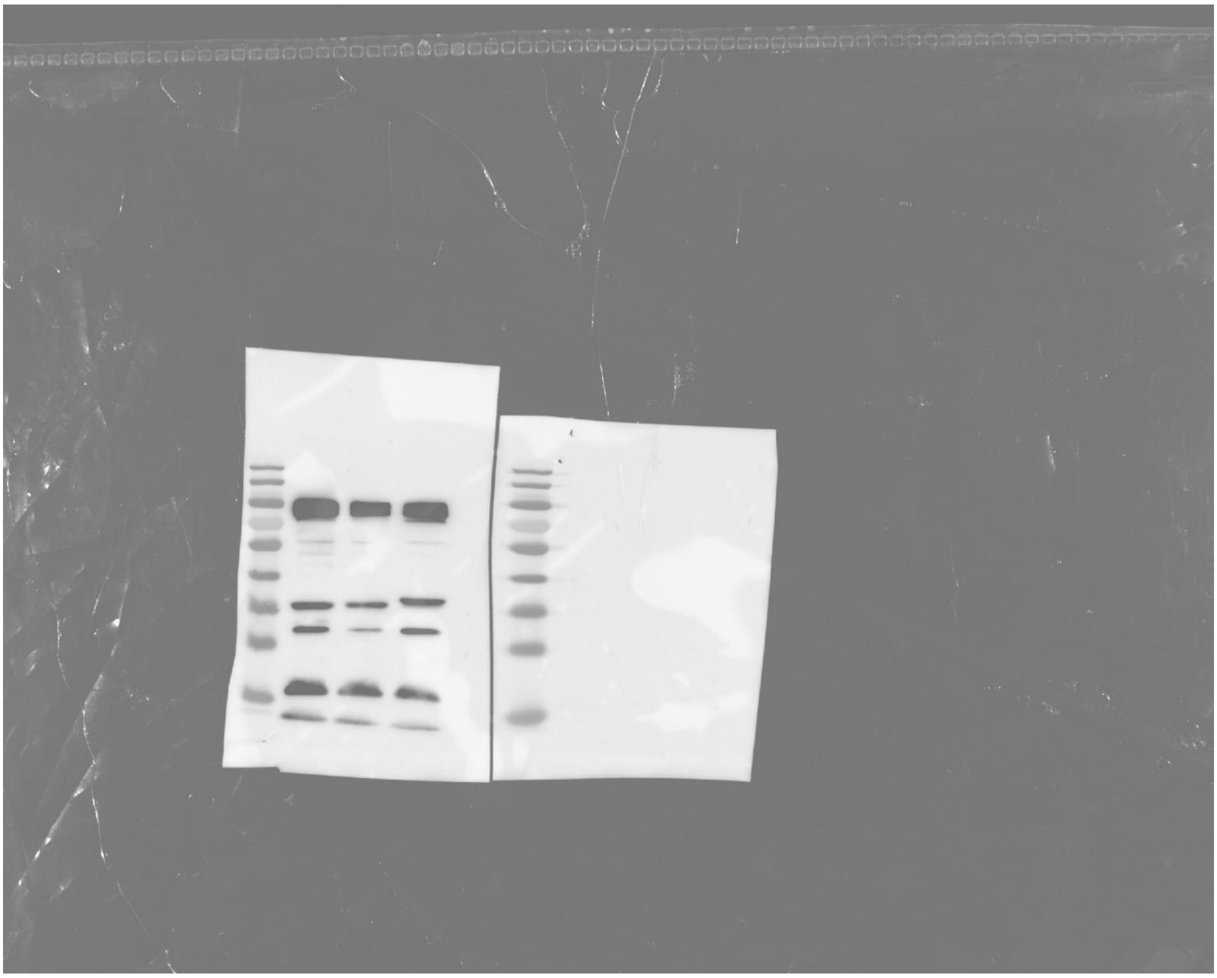

(G) Western blot of *E. coli* antigens, detection of anti-mouse IgG antibodies was performed in the serum of one immunized mouse and control (non-immunized) serum (n=4/group)

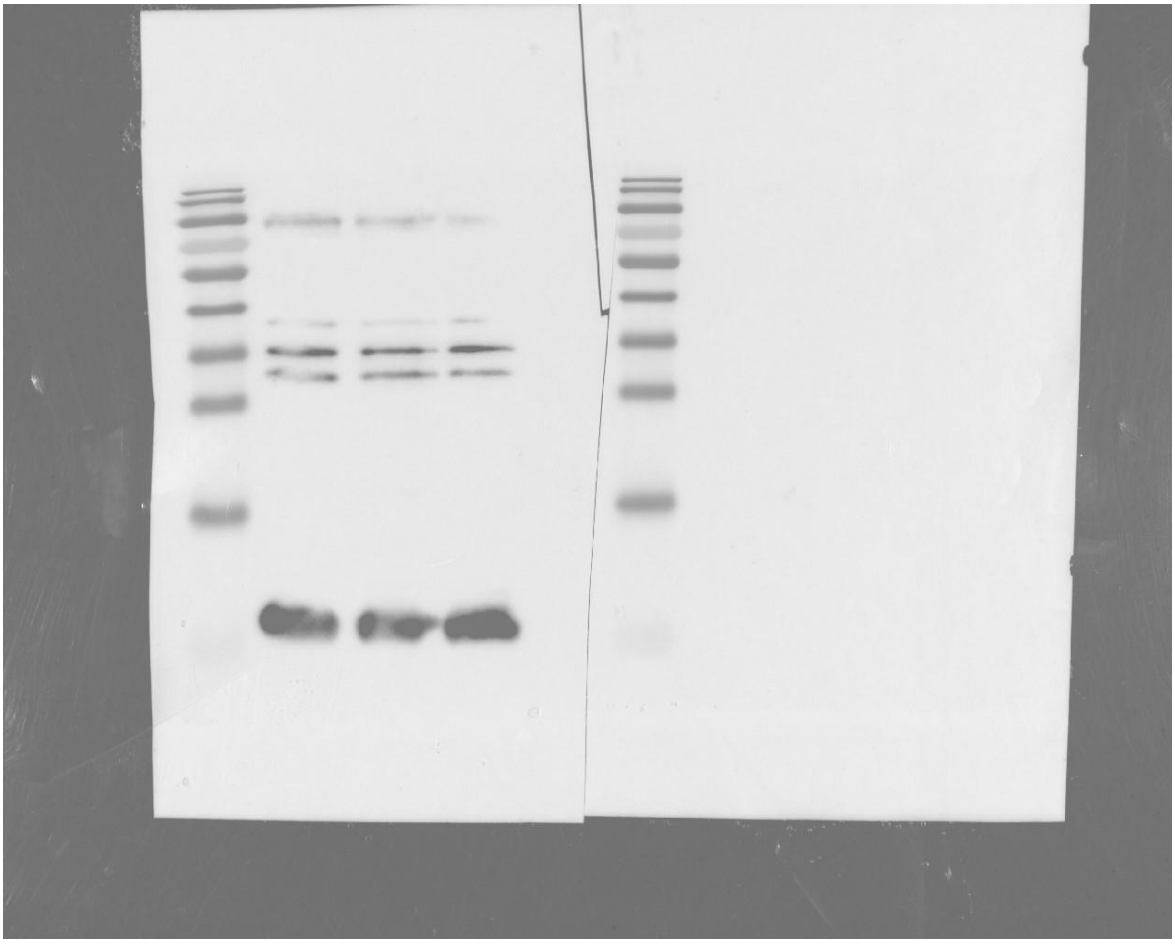

(H-I) Detection of polyclonal antibodies by Western Blot (H) serums of the pups' mice from immunized mothers and the presence of the control group (I) (serums of the pups mice from non-immunized mothers) (n=4/group).
