# Supplementary material for: A live attenuated vaccine to prevent severe neonatal Escherichia coli K1 infections
Source: Nat Commun. 2024 Apr 8;15:3021. doi: 10.1038/s41467-024-46775-x (PMC11001983; doi:10.1038/s41467-024-46775-x)
Supplement: Supplementary file 3 — Reporting Summary [file 41467_2024_46775_MOESM3_ESM.pdf]

nature portfolio

Corresponding author(s): DAVID SKURNIK

Last updated by author(s): 23/12/26

Reporting Summary

Nature Portfolio wishes to improve the reproducibility of the work that we publish. This form provides structure for consistency and transparency in reporting. For further information on Nature Portfolio policies, see our Editorial Policies and the Editorial Policy Checklist.

Statistics

For all statistical analyses, confirm that the following items are present in the figure legend, table legend, main text, or Methods section.

n/a	Confirmed	
☐	☒	The exact sample size (n) for each experimental group/condition, given as a discrete number and unit of measurement
☐	☒	A statement on whether measurements were taken from distinct samples or whether the same sample was measured repeatedly
☐	☒	The statistical test(s) used AND whether they are one- or two-sided<br>*Only common tests should be described solely by name; describe more complex techniques in the Methods section.*
☐	☒	A description of all covariates tested
☐	☒	A description of any assumptions or corrections, such as tests of normality and adjustment for multiple comparisons
☐	☒	A full description of the statistical parameters including central tendency (e.g. means) or other basic estimates (e.g. regression coefficient) AND variation (e.g. standard deviation) or associated estimates of uncertainty (e.g. confidence intervals)
☐	☒	For null hypothesis testing, the test statistic (e.g. F, t, r) with confidence intervals, effect sizes, degrees of freedom and P value noted<br>*Give P values as exact values whenever suitable.*
☒	☐	For Bayesian analysis, information on the choice of priors and Markov chain Monte Carlo settings
☐	☐	For hierarchical and complex designs, identification of the appropriate level for tests and full reporting of outcomes
☒	☐	Estimates of effect sizes (e.g. Cohen's d, Pearson's r), indicating how they were calculated

Our web collection on statistics for biologists contains articles on many of the points above.

Software and code

Policy information about availability of computer code

Data collection	No sofware was used
Data analysis	RNA sequencing data using CLC Genomics Workbench 24, HEC software for cytometry , GraphPad Prism software (v 9, GraphPad Software LLC, San Diego, CA, USA)

For manuscripts utilizing custom algorithms or software that are central to the research but not yet described in published literature, software must be made available to editors and reviewers. We strongly encourage code deposition in a community repository (e.g. GitHub). See the Nature Portfolio guidelines for submitting code & software for further information.

Data

Policy information about availability of data

All manuscripts must include a data availability statement. This statement should provide the following information, where applicable:

- Accession codes, unique identifiers, or web links for publicly available datasets
- A description of any restrictions on data availability
- For clinical datasets or third party data, please ensure that the statement adheres to our policy

The authors state that ail the data is available, including the data sources.
https://www.ncbi.nlm.nih.gov/geo/query/acc.cgi?acc=GSE252194

Research involving human participants, their data, or biological material

Policy information about studies with human participants or human data. See also policy information about sex, gender (identity/presentation), and sexual orientation and race, ethnicity and racism.

Reporting on sex and gender	No human data were used in this manuscript.
Reporting on race, ethnicity, or other socially relevant groupings	No human data were used in this manuscript.
Population characteristics	No human data were used in this manuscript.
Recruitment	No human data were used in this manuscript.
Ethics oversight	No human data were used in this manuscript.

Note that full information on the approval of the study protocol must also be provided in the manuscript.

Field-specific reporting

Please select the one below that is the best fit for your research. If you are not sure, read the appropriate sections before making your selection.

☒ Life sciences ☐ Behavioural & social sciences ☐ Ecological, evolutionary & environmental sciences

For a reference copy of the document with all sections, see nature.com/documents/nr-reporting-summary-flat.pdf

Life sciences study design

All studies must disclose on these points even when the disclosure is negative.

Sample size	We used groups of 4 mice per group for reasons of animal experimentation ethics in our country. N=4 per group. In addition, several groups of 4 adult mice and baby mice were used. We used both statistical test : Mantel–Haenszel log-rank test & Mann-Whitney non-parametric test. No sample size calculation was carried out. Sample sizes were chosen in accordance with current animal experimentation rules. All statistical tests used are mentioned in the manuscript.
Data exclusions	No data excluded
Replication	The experiments were carried out 3 times. And the experiments were all reproducible.
Randomization	No randomization. We used female mouse models to look at the impact of maternai vaccination on the protection of baby mice against E. coli meningitis. We created two groups: the experimental group and the control group. These groups are created by dividing the sample into two distinct groups using random assignment to avoid bias. As a result, these groups are identical, except that the experimental group receives the treatment being tested while the control group does not. Control groups allow the results of two identical groups to be compared so that any changes identified between the two groups can be attributed to the treatment administered.
Blinding	We created two groups: the experimental group and the control group. These groups are created by dividing the sample into two distinct groups using random assignment to avoid bias. These groups are therefore identical, except that the experimental group receives the treatment being tested while the control group does not. Control groups allow the results of two identical groups to be compared so that any changes identified between the two groups can be attributed to the treatment administered. We were not blinded to the allocation of groups during data collection and/or analysis because we composed the groups ourselves. The data from both groups were treated in the same way without bias.

Reporting for specific materials, systems and methods

We require information from authors about some types of materials, experimental systems and methods used in many studies. Here, indicate whether each material, system or method listed is relevant to your study. If you are not sure if a list item applies to your research, read the appropriate section before selecting a response.

Materials & experimental systems

n/a	Involved in the study
☐	☒ Antibodies
☐	☒ Eukaryotic cell lines
☒	☐ Palaeontology and archaeology
☐	☒ Animals and other organisms
☒	☐ Clinical data
☒	☐ Dual use research of concern
☒	☐ Plants

Methods

n/a	Involved in the study
☒	☐ ChIP-seq
☐	☒ Flow cytometry
☒	☐ MRI-based neuroimaging

Antibodies

Antibodies used

Anti-mouse secondary antibodies (rabbit antI-mouse lgG H&L, Abcam, Paris, France),
CD45 PerCP-Cy5.5 Biolegend 103132
CD19 APCeF780 eBioscie nce 47-0193-80
TCRb AF700 Biolegend 109224 CD4 PB Biolegend 100534
CDS BV605 BD Horizon 563152
FOXP3 APC lnVit rogen 17-5773-82.
All primary and secondary antibodies (with supplier name, catalogue number, clone name and batch number where applicable) are listed here in the report summary and referenced in the manuscript/supplementary table. In addition, all antibodies listed here are also provided in the manuscript (with dilution).

Validation

The valid at ion of the antibodies was based on the manufacturer's validation on their site.
https:// www.abcam .com/produ cts/secon dary-antibodies/ra bbit-mouse-igg-hl-ab6709.htm..
Rabbit Anti-Mouse IgG H&LSee all IgG secondary antibodies, Host species : Rabbit ; Target species : Mouse. Tested applications : Suitable for: WB, IP, Immunodiffusion, Conjugation, ICC/IF, ELISA, IHC-P, IHC-Frmore details. Immunogen : Mouse IgG, whole molecule

https:// www.b iolegend.corn/fr-ch/soluble-mhc/percp-cya nine5-5-a nti-mouse-cd45 2-antibody-4 271 ?GrouplD=BLG1934 https:// www.thermofisher .corn/a ntibody/product/CD19-Antibody-cl one-eBio1D3-l D3-Monoclona 1/47-0193-82
Isotype Control : PerCP/Cyanine5.5 Rat IgG2b, κ Isotype Ctrl
Verified Reactivity : Mouse
Antibody Type : Monoclonal
Host Species : Rat
Immunogen : Mouse thymus or spleen
Formulation : Phosphate-buffered solution, pH 7.2, containing 0.09% sodium azide.
Preparation : The antibody was purified by affinity chromatography, and conjugated with PerCP/Cyanine5.5 under optimal conditions.
Concentration : 0.2 mg/ml
Storage & Handling : The antibody solution should be stored undiluted between 2°C and 8°C, and protected from prolonged exposure to light. Do not freeze.
Recommended Usage : Each lot of this antibody is quality control tested by immunofluorescent staining with flow cytometric analysis. For flow cytometric staining, the suggested use of this reagent is = 0.25 µg per 106 cells in 100 µl volume. It is recommended that the reagent be titrated for optimal performance for each application.

https:// www.bio legend.corn/en-gb/products/alexa-fluor- 700-anti-mouse-tcr-beta-chain-anti body-4537?Clone=H57-597 https:// www.bio legend.corn/de-at/soluble-mhc/pacific-bl ue-anti-mo use-cd4-anti body-2855 ?GrouplD=BLG42 l l
Alexa Fluor® 700 Armenian Hamster IgG Isotype Ctrl
Verified Reactivity :Mouse
Antibody Type : Monoclonal
Host Species : Armenian Hamster
Immunogen : Affinity purified TCR from mouse DO-11.10 cells
Formulation :Phosphate-buffered solution, pH 7.2, containing 0.09% sodium azide.
Preparation : The antibody was purified by affinity chromatography and conjugated with Alexa Fluor® 700 under optimal conditions.
Concentration : 0.5 mg/ml
Storage & Handling : The antibody solution should be stored undiluted between 2°C and 8°C, and protected from prolonged exposure to light. Do not freeze.
Recommended Usage :Each lot of this antibody is quality control tested by immunofluorescent staining with flow cytometric analysis. The suggested use of this reagent is ≤1.0 µg per million cells in 100 µl volume. It is highly recommended that the reagent be titrated for optimal performance for each application.

https://www.bdbiosciences.com/en-ie/products/reagents/flow-cytometry-reagents/research-reagents/single-color-antibodies-ruo/bv605-rat-anti-mouse-cd8a.563152
Alternative Name: Cd8a; CD8 alpha chain; Ly-2; Lyt2; Lyt-2; Ly-35; Ly-B
Reactivity: Mouse (QC Testing)
Isotype: Rat LOU, also known as Louvain, LOU/C, LOU/M IgG2a, κ
Immunogen: Mouse Spleen Cells or Thymocyte Membranes
Application: Flow cytometry (Routinely Tested)
Concentration: 0.2 mg/ml

RRID: AB_2738030
Storage Buffer: Aqueous buffered solution containing ≤0.09% sodium azide.
Regulatory Status:RUO

https://www.thermofisher .corn/a ntibody/product/FOXP3-Antibody-clone- FJK-16s-Monoc lona 1/17-5 773-82

Species Reactivity: Bovine, Dog, Cat, Mouse, Pig, Rat
Published species: Dog, Horse, Human, Mouse, Pig, Rat
Host/Isotype: Rat / IgG2a, kappa
Recommended Isotype Control: Rat IgG2a kappa Isotype Control (eBR2a), APC, eBioscience™
Class: Monoclonal
Type: Antibody
Clone: FJK-16s
Conjugate: APCView additional formats
Excitation/Emission Max: 651/660 nmView spectraspectra
Form:Liquid; Concentration: 0.2 mg/mL
Purification: Affinity chromatography
Applications
Tested Dilution
Publications
Immunohistochemistry (IHC), Immunohistochemistry (PFA fixed) (IHC (PFA)), Immunohistochemistry (Frozen) (IHC (F)), Immunocytochemistry (ICC/IF), Flow Cytometry (Flow), ChIP assay (ChIP),T-Cell Activation (TCA

Eukaryotic cell lines

Policy information about cell lines and Sex and Gender in Research

Cell line source(s)	Hela epithelia l cells ATCC<br>https://www.atcc.org/products/ccl-2
Authentication	https://www.atcc.org/products/ccl-2. No authentification
Mycoplasma contamination	PCR detection test: This test involves the speci fic PCR amplification of mycoplasma DNA. The kit developed by Sigma uses pairs of primers directed against the 16S RNA sequence, which is highly conserved, enabling most co mmonly encounte red mycoplasmas to be detected. It dete cts 19 different mycoplasma species. We confirm that the cell line tested negative for mycoplasma contamination
Commonly misidentified lines<br>(See ICLAC register)	No

Animals and other research organisms

Policy information about studies involving animals; ARRIVE guidelines recommended for reporting animal research, and Sex and Gender in Research

Laboratory animals	BALB/C female mice (Supplier January Lab) 6 months old. We have described the housing conditions for the mice in the manuscript.
Wild animals	No wild anima is used
Reporting on sex	We used only females in order to look at the impact of maternai vaccination on the protection of baby mice against neonatal E. co li Kl meningitis. Only female animal were used for the adult animal as the target of the vaccine is women with a pregnancy project. Both sex were used for baby mice
Field-collected samples	No samples were collected in the field.
Ethics oversight	The mouse experiments were conducted in accordance with local, nat ional and European Union guidelines and had been approved by the Animal Care and Use Committee at Université Paris Cité {Paris, France; reference: 2020011519022360; approval number A75-14-08).

Note that full information on the approval of the study protocol must also be provided in the manuscript.

Plants

Seed stocks

We didn't use any plants

Novel plant genotypes

We didn't use any plants

Authentication

We didn't use any plants

Flow Cytometry

Plots

Confirm that:

☒ The axis labels state the marker and fluorochrome used (e.g. CD4-FITC).

☒ The axis scales are clearly visible. Include numbers along axes only for bottom left plot of group (a 'group' is an analysis of identical markers).

☐ All plots are contour plots with outliers or pseudocolor plots.

☒ A numerical value for number of cells or percentage (with statistics) is provided.

Methodology

Sample preparation

Seven days after full immunizati on, BALB/c mice were sac rificed, and the spleens were col-lected for the flow cytometry ana lysis of lym phocytes. Red blood celis were removed from spleen cell suspensions by lysis with ammon ium chloride buffer. The remaining cells were then incubated with LIVE/DEAD ' M Fixable Aqua Dead Cell Stain Kit (lnvitrogen L34957, Thermo Scientific Inc., Branchburg , NJ, USA)Collect spleen in HBSS+10% SVF on iceCrush the spleen on a 40um cell sieve Wash 1 time with 5m l of cold HBSS + 10% SVF (300g centri 5' 4'C)Wash 1 time with 10ml HBSS (RT) (300g centri 5' 4'C)Discard the supernatant completely (empty the supernatant into a waste bin and remove the last microlitres of supernatant above the pellet with a pipette).Rub the tube on a rack to dislodge the pelletResuspend in 2m l of red cell lysis buffer (ACK buffer).

Seven days after full immunizati on, BALB/c mice were sac rificed, and the spleens were col-lected for the flow cytometry ana lysis of lym phocytes. Red blood celis were removed from spleen cell suspensions by lysis with ammon ium chloride buffer. The remaining cells were then incubated with LIVE/DEAD ' M Fixable Aqua Dead Cell Stain Kit (lnvitrogen L34957, Thermo Scientific Inc., Branchburg , NJ, USA)Collect spleen in HBSS+10% SVF on iceCrush the spleen on a 40um cell sieve Wash 1 time with 5m l of cold HBSS + 10% SVF (300g centri 5' 4'C)Wash 1 time with 10ml HBSS (RT) (300g centri 5' 4'C)Discard the supernatant completely (empty the supernatant into a waste bin and remove the last microlitres of supernatant above the pellet with a pipette).Rub the tube on a rack to dislodge the pelletResuspend in 2m l of red cell lysis buffer (ACK buffer). Gent ly resuspend with a pipette (do not vortex) for 300ml: NH4CI 2.48g + EDTA 0.SM 60ul + KHCO3 0.3g + HCI lN pH 7.2-7 .4+ H2O MQ QSP, filtered, stored at 4°CStart the timer as soon as the ACK is added and incubate for 5' in the dark at RTWash 2 times wit h 4ml HBSS-SVF10% (300g 5' 4°C), resuspend in 4ml HBSS-SVF10% .Pass the cells through a ce ll sieve (u se a FACS tube cell sieve as it has a tighter mesh)Count the cells: pre-dilute to 1/20 (Sul cells, 95 ul HBSS+l0%SVF) then dilut e to ½ in trypan blueAdd 2.5x106 cells per FACS tube(Make an extra tube that will not be labelled)

Instrument

A Fortessa fl ow cytometer (BD Biosciences, San Jose, CA, USA) was used to analyze 100000 stained ce lls. Doublets were excluded from the analysis by using appropriate forward scat-ter/side scatte r gates

Software

Data were analyzed with Flowjo software

Cell population abundance

Sampies	Total number of events	%living cells
Cl	1. 22 X 1000000	91,8
C2	l.09x 1000000	90,3
C3	l.29x 1000000	90,4
C4	l.2l x1000000	92,2
1	1.07x1000000	89,7
2	1.22x1000000	89,9
3	0.93x1000000	89,4
4	1.22x1000000	91,6

ColLect spleen in HBSS+10% SVF on iceCrush th e spleen on a 40um cell sieve Wash 1 t ime with 5ml of cold HBSS + 10% SVF (300g centri 5' 4°C)Wash 1 t ime with 10ml HBSS (RT) (300g centri 5' 4°C)Discard the supernatant completely (empty the supe rnat ant into a waste bin and remove the last microlitres of sup ernatan t above the pellet with a pip ette) .Rub th e tub e on a ra ck to dislodge the pellet Resusp end in 2ml of red cell lysis buffer (ACK buffe r). Gent ly resuspend with a pipette (do

not vortex) for 300ml : NH4CI 2.48g + EDTA 0.SM 60ul + KHCO3 0.3g + HCI lN pH 7.2-7.4 + H2O MQ QSP, filtered, stored at 4° CStart the t im er as soon as the ACK is added and incubate for 5' in the dark at RTWash 2 times with 4ml HBSS-SVF10% (300g 5' 4°(), resuspend in 4ml HBSS-SVF10%.Pass the cells through a cell sieve (use a FACS tube cell sieve as it has a tighter mesh)Count the cells: pre-dilute to 1/20 (Su l cells, 95ul HBSS+10%SVF) then dilute to ½ in trypan blueAdd 2.5x106 celis per FACS tube(Make an extra tube that will not be labelled)

Gating strategy

First of all, from all the events that pass, we select the cells. Then we select the single cells. Then I will select the li ving cells. After selecting the living cells I take the hematopoietic cells (so CD 45+). Then I look at the expr ession of CD19 and TCRb to sepa r ate B lymphocytes from T lymphocytes. In the T lymphocytes I look at the expr ession of CD8 and CD4 and in the CD4 1 lo o k at FOXP3 for regulatory T cells .

☒ Tick this box to confirm that a figure exemplifying the gating strategy is provided in the Supplementary Information.
